# Optimization of Evans blue quantitation in limited rat tissue samples

**DOI:** 10.1038/srep06588

**Published:** 2014-10-10

**Authors:** Hwai-Lee Wang, Ted Weita Lai

**Affiliations:** 1 Graduate Institute of Clinical Medical Science, China Medical University, Taichung 404, Taiwan; 2 Translational Medicine Research Center, China Medical University Hospital, Taichung 404, Taiwan

## Abstract

Evans blue dye (EBD) is an inert tracer that measures plasma volume in human subjects and vascular permeability in animal models. Quantitation of EBD can be difficult when dye concentration in the sample is limited, such as when extravasated dye is measured in the blood-brain barrier (BBB) intact brain. The procedure described here used a very small volume (30 µl) per sample replicate, which enabled high-throughput measurements of the EBD concentration based on a standard 96-well plate reader. First, ethanol ensured a consistent optic path length in each well and substantially enhanced the sensitivity of EBD fluorescence spectroscopy. Second, trichloroacetic acid (TCA) removed false-positive EBD measurements as a result of biological solutes and partially extracted EBD into the supernatant. Moreover, a 1:2 volume ratio of 50% TCA ([TCA final] = 33.3%) optimally extracted EBD from the rat plasma protein-EBD complex *in vitro* and *in vivo*, and 1:2 and 1:3 weight-volume ratios of 50% TCA optimally extracted extravasated EBD from the rat brain and liver, respectively, *in vivo*. This procedure is particularly useful in the detection of EBD extravasation into the BBB-intact brain, but it can also be applied to detect dye extravasation into tissues where vascular permeability is less limiting.

Evans blue dye (EBD)^1^ is a commonly used tracer for the estimation of plasma volume in humans and the study of vascular permeability in animal models. Similar to other dyes used in human diagnosis^1^,^2^,^3^, EBD is non-toxic and not metabolically active in mammalian circulation^1^,^4^. Because of its rapid binding to serum albumin^5^ and lack of cellular uptake^6^, its plasma concentration remains relatively constant within the hours following intravenous injection^4^,^7^,^8^,^9^,^10^,^11^,^12^. Therefore, its final plasma concentration following a brief moment of circulatory distribution is used to depict the total plasma volume of test subjects, including human patients^4^,^9^,^10^,^13^,^14^,^15^. In addition, its extravasation into central and peripheral organs following a more prolonged time period correlates with vascular leakage of serum albumin, and its leakage into the brain parenchyma indicates blood-brain barrier (BBB) disruption^11^,^16^,^17^,^18^,^19^,^20^.

EBD extraction prior to spectroscopic measurements can substantially improve the accuracy of dye quantitation. In addition to direct interference by the inherent turbidity and spectroscopic properties of lipids and proteins^6^,^8^,^21^, EBD binding to these biological solutes can directly alter the spectroscopic property of the dye^22^. Therefore, EBD extraction is particularly crucial when the EBD concentration in a tissue sample is low compared with the concentration of biological solutes. For example, the determination of the EBD amount that is extravasated into the BBB-intact or minimally BBB-damaged brain is greatly hindered because of (1) the high content of biological solutes present in brain tissues and (2) the poor permeability of EBD across the BBB^11^,^16^,^17^,^18^,^19^,^20^. Trichloroacetic acid (TCA), which can compete with EBD for binding to plasma proteins^23^, is a reagent commonly used to extract EBD^20^,^24^,^25^,^26^,^27^,^28^,^29^,^30^,^31^,^32^,^33^. Despite its wide application in research, the percentage yield of EBD that can be extracted via TCA remains elusive, and no report has aimed to optimize its use.

The present study comprehensively evaluated the efficiency of the TCA method in the extraction of EBD from plasma and brain tissue *in vitro* and *in vivo*. An optimized protocol in which EBD was extracted from limited biological samples and measured with high-throughput spectroscopy based on a standard 96-well plate reader is described. The relevance of the findings to vascular permeability studies in general and BBB permeability studies in particular are discussed.

## Results

### Spectroscopic Readings of Standard EBD Solutions versus False-Positive Readings from Biological Solutes

**Figure 1:**
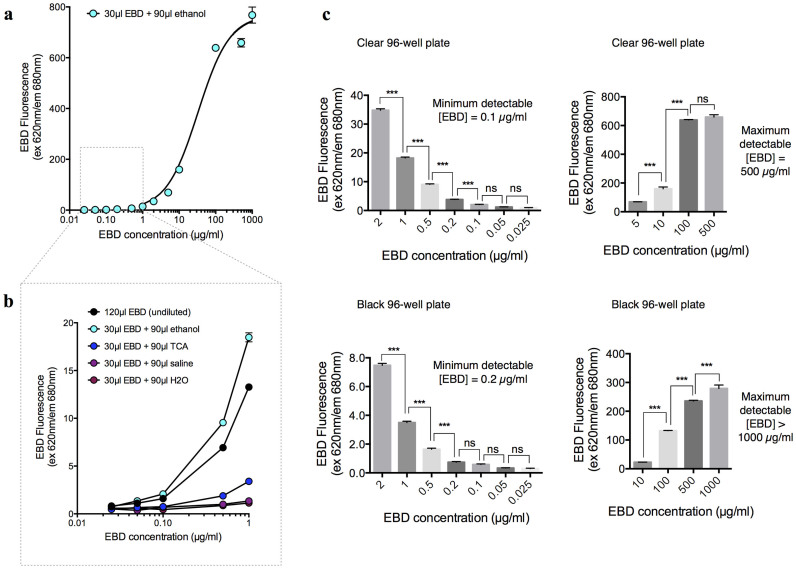
Evans blue dye (EBD) fluorescence (620 nm excitation/680 nm emission) detection. Standard curve showing EBD fluorescence as a function of dye concentration in clear (a–c) and black (c) 96-well plates. (a) Full EBD standard curve based on a 1:3 ethanol dilution. Each 30 µl replicate of EBD (in 50% TCA/0.9% saline) was supplemented with 90 µl of 95% ethanol to ensure consistent optic path length. Concentrations of the 30 µl replicates are indicated on the x-axis. (b) EBD standard curves for the 120 µl undiluted dye (in 50% TCA/0.9% saline) and 30 µl dye (in 50% TCA/0.9% saline) diluted to 120 µl with one solvent: 95% ethanol, 50% TCA, 0.9% saline, or water. Concentrations of the 120 µl undiluted dye and the 30 µl dye (prior to dilution) are indicated on the x-axis. (c) The maximum and minimum detectable EBD concentrations in the clear and black 96-well plates were determined by Tukey's multiple comparisons tests. A significant difference (***P<0.001) indicates a detectable concentration difference, and no significance (ns; P>0.05) indicates that the concentration difference was not detectable.

The concentration range within which EBD can be accurately measured by its fluorescence spectroscopy was compared with the false-positive readings that could result from auto-fluorescence of biological samples. A 96-well plate was selected for spectroscopy because of its wide commercial availability; furthermore, it provides a reasonable optic path length even with a limited raw sample. EBD fluorescence exhibited a concentration-dependent increase from 0.1-100 µg/ml (*n* = 12 per group) (Fig. 1a). A step-wise comparison identified 0.1 µg/ml as the lowest detectable concentration with the clear 96-well plates and 0.2 µg/ml as the lowest detectable concentration with the black 96-well plates (*n* = 12 per group) (Fig. 1c). Thus, the clear 96-well plate was more sensitive for the quantification of a limited sample and was used in the remainder of the study. Given the objective to minimize the samples required for accurate quantitation, we added only 30 µl of EBD standard solution to each well. To ensure a consistent optic path length for each measurement, each well was subsequently filled with an additional 90 µl of ethanol, TCA, saline, or water. Notably, increasing the optic path length with ethanol, compared with other solvents, substantially improved the sensitivity of fluorescence spectroscopy (*n* = 12 per group; *P* < 0.0001) (Fig. 1b). Thus, the remaining spectroscopic assays in this study used 90 µl ethanol to increase the optic path length in each well of the 30 µl samples.

**Figure 2:**
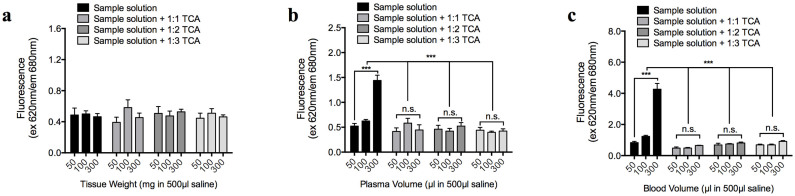
False-positive fluorescence readings (620 nm excitation/680 nm emission) from biological solutes. (a–c) Auto-fluorescence of biological solutes from the brain (a), blood (b), and plasma (c) samples (diluted to 500 µl in saline), with or without protein-precipitation by 1:1–1:3 50% trichloroacetic acid (TCA). [TCA _final_] following the addition of 1:1, 1:2, and 1:3 volume ratios of 50% TCA were 25, 33, and 37.5%, respectively.

The brain tissue extract supernatants did not appear to auto-fluoresce at 620 nm/680 nm (*n* = 5 per group) (Fig. 2a); however, the plasma and blood samples strongly auto-fluoresced at 620 nm/680 nm when 300 µl were diluted to 500 µl in saline (*n* = 5 per group; *P* < 0.0001) (Fig. 2b, c). Nevertheless, the precipitation of biological solutes from the plasma and blood samples via TCA removed their auto-fluorescence almost entirely (*n* = 5 per group; *P* < 0.0001) (Fig. 2b, c). When the data from Fig 2a–c and 1a were combined, auto-fluorescence from the plasma and whole blood samples could generate up to 0.05–0.06 and 0.2–0.3 µg/ml of false-positive EBD readings, respectively, which were removed by TCA-mediated precipitation.

**Figure 3:**
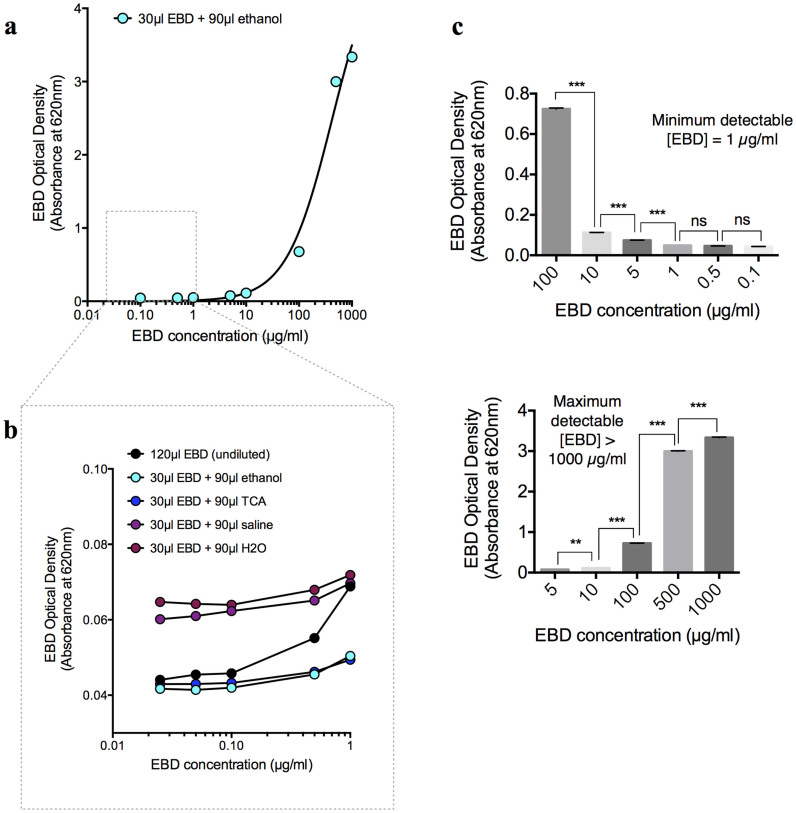
Evans blue dye (EBD) optical density (absorbance of 620 nm) detection. Standard curve showing EBD absorbance as a function of dye concentration. (a) Full EBD standard curve based on a 1:3 ethanol dilution. Each 30 µl replicate of EBD (in 50% TCA/0.9% saline) was supplemented with 90 µl of 95% ethanol to ensure consistent optic path length. Concentrations of the 30 µl replicates are indicated on the x-axis. (b) EBD standard curves for the 120 µl undiluted dye (in 50% TCA/0.9% saline) and 30 µl dye (in 50% TCA/0.9% saline) diluted to 120 µl with one solvent: 95% ethanol, 50% TCA, 0.9% saline, or water. Concentrations of the 120 µl undiluted dye and the 30 µl dye (prior to dilution) are indicated on the x-axis. (c) The maximum and minimum detectable EBD concentrations in the clear 96-well plate were determined by Tukey's multiple comparisons tests. A significant difference (***P<0.001 and **P<0.01) indicates a detectable concentration difference, and no significance (ns; P>0.05) indicates that the concentration difference was not detectable.

**Figure 4:**
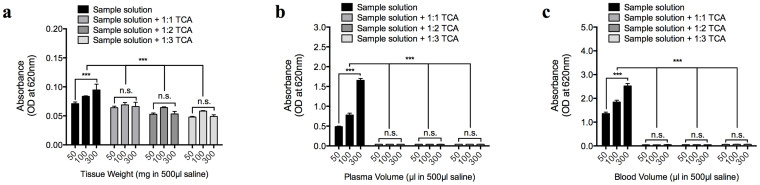
False-positive optical density readings (absorbance of 620 nm) from biological solutes. (a–c) Absorbance of biological solutes from the brain (a), blood (b), and plasma (c) samples (diluted to 500 µl in saline), with or without protein-precipitation by 1:1–1:3 of 50% trichloroacetic acid (TCA). [TCA _final_] following the addition of 1:1, 1:2, and 1:3 volume ratios of 50% TCA were 25, 33, and 37.5%, respectively.

The concentration range within which EBD can be measured from its optical density (absorbance of 620 nm light) and the false-positive readings that could result from the optical density of biological solutes were also compared. An EBD absorbance of 620 nm light exhibited a concentration-dependent increase between 1–1000 µg/ml (*n* = 12 per group), with an empirically determined molar extinction coefficient of 7.2 M^−1^ cm^−1^, which is consistent with previously published values^34^ (Fig. 3a, c). Therefore, in comparison with the aforementioned fluorescence readings, the absorbance readings may be more suitable for the measurement of EBD concentrations greater than 100 µg/ml. Unlike the EBD fluorescence-concentration curve in which ethanol substantially increased the detection threshold, none of the solvents used to increase the optic path length increased the slope of the EBD concentration-absorbance standard curve (*n* = 12 per group) (Fig. 3b). Without TCA treatment, the biological extracts (0.9% saline) from the brain, blood, or plasma produced strong absorbance readings at 620 nm (*n* = 5 per group; *P* < 0.0001) (Fig. 4a–c), thereby producing up to 400–500 µg/ml of false-positive EBD readings. The precipitation of these solutes via TCA removed the tissue absorbance (*n* = 5 per group; *P* < 0.0001) (Fig. 4a–c) and thereby avoided false-positive EBD readings.

### Percent Yield of EBD Extracted from *in vitro* Protein Complex and *in vivo* Biological Samples with Different Volume Ratios of TCA

**Figure 5:**
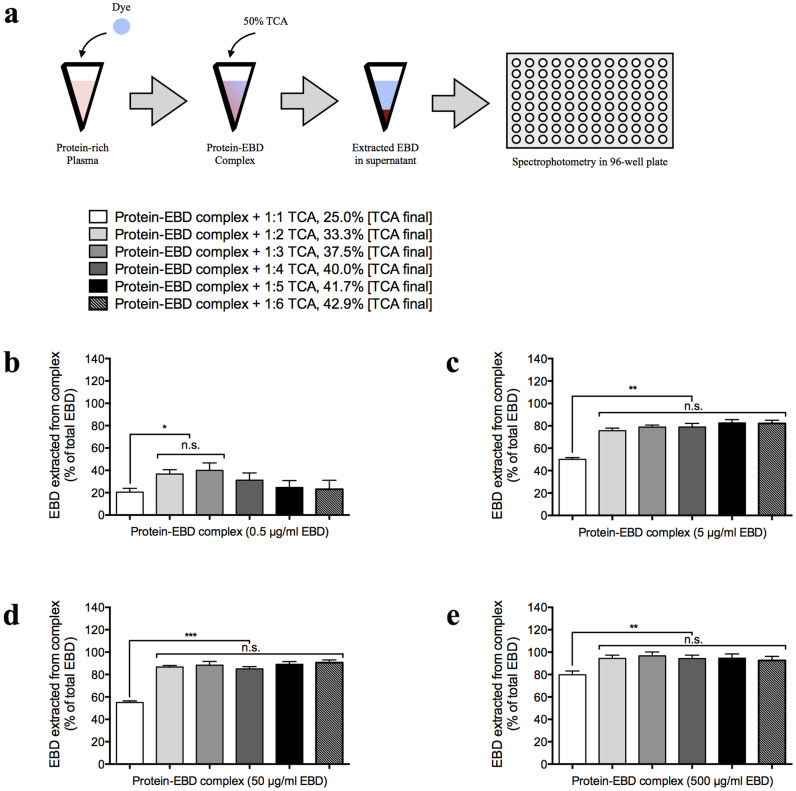
Extraction of Evans blue dye (EBD) from the protein-EBD complex *in vitro*. (a) Illustration showing the experimental protocol for (b–e). EBD formed a complex with plasma proteins. Thereafter, the dye was extracted from the protein with 50% trichloroacetic acid (TCA), and its concentration was detected by spectroscopy in a 96-well plate. (b–e) The percentage yields of EBD that were extracted from the protein-EBD complex by different volume-ratios of 50% TCA (in 0.9% saline), which resulted in different [TCA _final_], were compared. The extracted EBD was measured by spectroscopy and normalized to the total EBD added to the plasma samples to generate the protein-EBD complex solutions. The total EBD concentrations were as follows: 0.5 µg/ml (b), 5 µg/ml (c), 50 µg/ml (d), and 500 µg/ml (e).

We investigated the percentage of EBD that can be extracted from the protein-EBD complex via TCA and the optimal volume ratio of a 50% TCA solution used to extract EBD. A protein-rich plasma solution was obtained from the arterial blood and mixed with an equal volume of EBD in 0.9% saline to generate the protein-EBD complex (see Fig. 5a for illustration). To extract the EBD from the newly formed protein complex, each sample was subsequently mixed with different volume ratios of 50% TCA, and the concentration of the extracted EBD solution was measured using spectroscopy. Here, 1:2–1:3 volume ratios of 50% TCA extracted only ~40% of the 0.5 µg/ml EBD from the protein-EBD complex solution (*n* = 6 per group; *P* < 0.05), and 1:4–1:6 volume ratios of 50% TCA diluted the sample too much such that EBD could not be detected (*n* = 6 per group) (Fig. 5b). In comparison, 1:2–1:6 volume ratios of 50% TCA extracted ~80, ~90, and ~100% of the 5, 50, and 500 µg/ml EBD, respectively, from the protein-EBD complex solutions (*n* = 6 per group; *P* < 0.01) (Fig. 5c–e).

**Figure 6:**
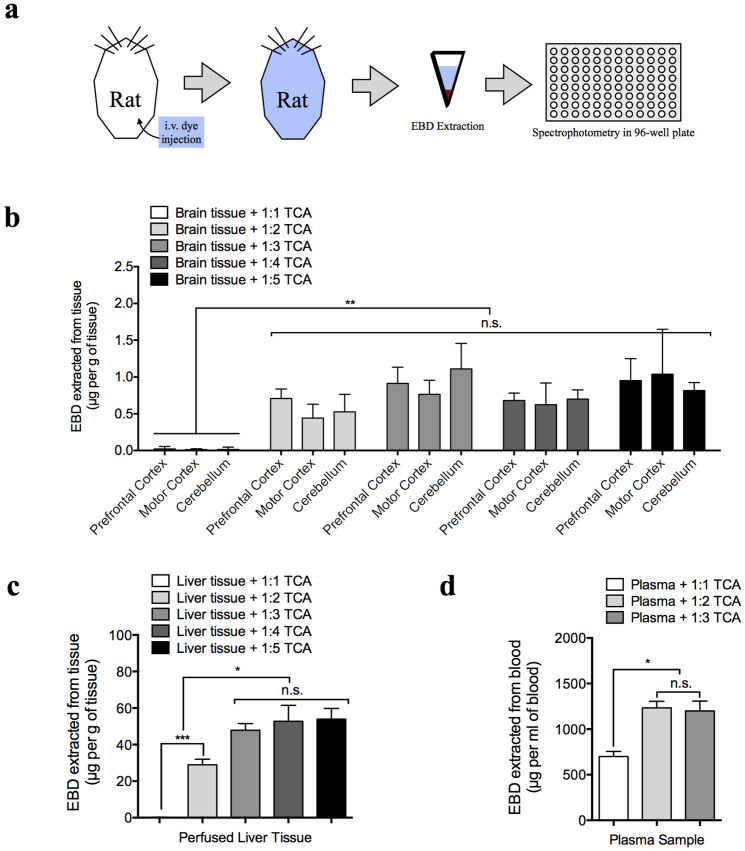
Extraction of Evans blue dye (EBD) from the protein-EBD complex and peripheral/central organs *in vivo*. (a) Illustration showing the experimental protocol for (b–d). Each rat was injected with EBD; EBD was extracted from the central/peripheral organs 2 h later, and its concentration was measured by spectroscopy in a 96-well plate. (b–d) EBD was extracted by different volume-ratios of 50% TCA (in 0.9% saline) from the brain parenchyma (b), liver parenchyma (c), and plasma (d) in rats injected with the dye 2 h prior to sample collection.

The optimal volume ratios of TCA that extracted extravasated EBD from the brain, liver, and blood of the rats intravenously injected with the dye *in vivo* were determined. The blue color of EBD provides an excellent contrast to the red color of blood, and successful intravenous injection in albino laboratory animals is immediately evident by the blue coloration of densely vascularized tissues (see Fig. 6a for illustration). Consistent with the minimal extravasation of EBD into the brain parenchyma, only ~1 µg/g (EBD/tissue weight) could be detected, and this amount was optimally extracted with 1:2–1:5 volume ratios of 50% TCA (*n* = 4 per group; *P* < 0.01) (Fig. 6b). In comparison, ~50 µg/g could be detected in the liver parenchyma; this amount was substantially extracted with a 1:2 volume ratio of 50% TCA (*n* = 4 per group; *P* < 0.0001) and most optimally extracted with 1:3–1:5 volume ratios of 50% TCA (*n* = 4 per group) (Fig. 6c). EBD primarily resided in the blood, with the plasma EBD concentration up to ~1300 µg/ml (*n* = 4 per group) (Fig. 6d). This amount was substantially extracted with a 1:1 volume ratio of 50% TCA and most optimally extracted with 1:2 or 1:3 volume ratios of 50% TCA (*n* = 4 per group; *P* < 0.05) (Fig. 6d).

## Discussion

The present study investigated the potential pitfalls associated with EBD detection and established an optimal procedure to extract and quantify EBD from limited biological samples. While these advancements may not be crucial when an ample sample for plasma volume determination can be obtained from human subjects^6^,^35^, these findings are essential when the raw sample is limited, such as when working with small laboratory animals or when the sample tissue has a low capacity for EBD extravasation, such as the BBB-intact brain parenchyma^11^,^16^,^17^,^18^,^19^. First, only a small volume of extracted sample (30 µl) is needed for EBD detection. Each sample replicate, which was added into the 96-well plate, was supplemented with a 3x volume of ethanol to ensure consistent optical path length. Ethanol was selected for this purpose because it substantially improved the sensitivity of EBD fluorescence spectroscopy. Second, the range within which EBD can be accurately quantified under this protocol was defined. The EBD maximally absorbs light of 620 nm and emits red fluorescence of 680 nm^17^,^18^; thus, its concentration in tissues and plasma can be determined by either parameter^6^,^18^,^35^. We have shown that EBD can be accurately determined from its fluorescence within a concentration range of 0.1–100 µg/ml and its absorbance within a range of 1->1000 µg/ml. In practice, a lower sensitivity of absorbance readings is sufficient for plasma volume studies in human subjects, where a large amount of blood can be obtained per subject^6^,^35^. In rodent studies of vascular permeability, the limitation of tissue mass would necessitate the higher sensitivity of fluorescent readings.

The common assumption that TCA completely extracts EBD (~100% yield) from the albumin-complex, thereby enabling the linear quantitation of dye concentration, was based on previous extraction experiments that used impractically high concentrations of over-saturating EBD (1000 ~ 4000 µg/ml) to generate plasma protein-EBD complexes^32^. However, these concentrations have never been identified in the animal brain, even following BBB disruption^33^. The present study reproduced these findings in which 500 µg/ml EBD was fully extracted from the plasma protein-EBD complex; however, it was also determined that any decrease in EBD concentration in the protein-EBD complex solution substantially decreased the percentage yield of the extraction.

In conclusion, in the previous two decades, there has been an increased interest in the use of EBD to study BBB permeability in small rodents. The TCA extraction technique has emerged as a popular technique for the extraction of EBD from the protein-EBD complex. The present study investigated the efficiency and pitfalls of this procedure and established an optimal protocol that will be of general interest to individuals who utilize EBD.

## Methods

### Preparation of standardized EBD solutions

Standard EBD solutions of various concentrations (0.025–1000 µg/ml) were prepared by dissolving dye powder (Sigma cat.# E2129-50G) in 50% TCA (diluted in 0.9% saline). To generate standard curves for use with small sample volumes, each replicate contained only 30 µl of standard solution in each well of a black or clear 96-well plate, which was subsequently supplemented with 90 µl of solvent to provide consistent optical path length for spectroscopic measurements. For comparison, the solvent used comprised 95% ethanol, 50% TCA (in 0.9% saline), 0.9% saline, or distilled water. A 120 µl undiluted EBD standard was also included to control for the dilution by the solvent. Each well was thoroughly mixed by repetitive pipetting, and the spectroscopic detection of (1) absorbance at 620 nm and (2) fluorescence by 620 nm excitation and 680 nm emission was conducted using a standard plate reader (SpectraMax® M2e). The maximum and minimum detectable concentrations were determined by a Tukey's multiple comparisons test and are indicated by a significant difference in the spectroscopic readings between each incremental increase in concentration (Fig. 1c). Thus, the minimum detectable concentrations for EBD fluorescence were 0.1 and 0.2 µg/ml for the clear and black 96-well plates, respectively. The maximum concentrations were 500 and >1000 µg/ml for the clear and black 96-well plates, respectively. The minimum concentration for absorbance detection was 1 µg/ml, and the maximum concentration was >1000 µg/ml.

### Biological solutes from brain, blood, and plasma

Three rats were used in these *in vitro* experiments. Each rat was euthanized by an overdose of urethane anesthesia, and its right femoral artery was cannulated to facilitate arterial blood collection. The blood samples were maintained on ice to slow coagulation (as no anti-coagulant was used during blood collection) and were centrifuged at 10,000 × *g* (for 20 min) to obtain a cell-free plasma sample. Prior to brain tissue isolation, each rat was thoroughly perfused with 0.9% saline to rid the brain of circulating blood. The isolated brain parenchyma was cut and weighed into small pieces (50, 100, and 300 mg) and incubated in 500 µl of 0.9% saline for at least 60 min to enable the soluble biomolecules to dissolve. The solutions were centrifuged at 10,000 × *g* for 10 min to sediment the non-dissolved tissue parts, and the supernatants that contained the brain solutes received no further treatment or were treated with 1:1, 1:2, or 1:3 volume-ratios of 50% TCA, which resulted in a [TCA_final_] of 25.0, 33.3, and 37.5%, respectively. The blood and plasma samples (50, 100, 300 µl) were each diluted with 0.9% saline to a final volume of 500 µl and received no further treatment or were treated with 1:1, 1:2, or 1:3 volume-ratios of 50% TCA, which resulted in a [TCA_final_] of 25.0, 33.3, and 37.5%, respectively. To prevent blood coagulation, the blood samples were treated with 0.25 U heparin (10 µl of 25 U heparin into a 1000 µl blood sample) immediately prior to dilution with 0.9% saline. Following TCA treatment, the brain, blood, and plasma samples were further centrifuged at 10,000 × *g* for 20 min to remove precipitated biomolecules. The final supernatant, with or without the TCA extraction procedure, was added to a clear 96-well plate (30 µl per well). Following the supplementation of each well with 90 µl of 95% ethanol to increase its optic path length, each well was thoroughly mixed by repetitive pipetting, and its absorbance (620 nm) and fluorescence (620 nm/680 nm) were determined.

### Preparation and extraction of the plasma protein-EBD complex

The plasma samples, which were collected as previously described, were mixed with equal volumes of EBD solutions (1, 10, 100, or 1000 µg/ml in 0.9% saline). Following at least 10 min to enable adequate plasma protein-EBD complex formation and with final EBD concentrations of 0.5, 5, 50, or 500 µg/ml, each sample was treated with different volume-ratios of 50% TCA (1:1–1:6, which resulted in a [TCA_final_] of 25.0, 33.3, 37.5, 40.0, 41.7, and 42.9%, respectively). The TCA-treated solutions were further centrifuged at 10,000 × *g* for 20 min to remove the precipitated plasma proteins and lipids, and the extracted supernatants were added to a clear 96-well plate (30 µl per well, supplemented with 90 µl of 95% ethanol and mixed thoroughly by pipetting) for the spectroscopic assay. Based on our standard curves in Fig. 1A and 3A, the concentrations of the extracted EBD from the 0.5 and 5 µg/ml samples could be accurately determined from their fluorescence (620 nm/680 nm) readings, and the concentrations from the 50 and 500 µg/ml samples could be determined from their absorbance (620 nm) readings.

### Calculation of extraction efficiency

The extraction efficiency was determined from the ratio of the extracted EBD concentration ([EBD_extracted_] in µg/ml) to the initial EBD concentration ([EBD_initial_] of 0.5, 5, 50, or 500 µg/ml). The [EBD_extracted_] was calculated by multiplying the measured EBD concentration, based on the standard curves derived from the spectroscopic readings, by the respective dilution factors that resulted from TCA treatment. Thus, the extraction efficiency was 100% when the [EBD_extracted_] was equal to the [EBD_initial_], and the extraction was only partial when the [EBD_extracted_] was less than the [EBD_initial_].

### *In vivo* EBD injection and dye extraction

Eight rats were used for the in vivo experiments, which were performed under deep urethane anesthesia (4 g/kg, i.p.). Each rat was placed on a heating pad to maintain body temperature throughout the experiment. The right femoral vein and artery were cannulated. EBD, which was prepared as a 4% solution in 0.9% saline, was injected as a single bolus dose of 2 ml/kg via the venous cannula. After a 120 min period to enable uniform blood distribution, the EBD-stained blood was collected from the arterial cannula. The blood samples were placed on ice until centrifugation at 10,000 × *g* to sediment blood cells, and the EBD-stained plasma supernatants were collected in separate tubes. EBD extraction from plasma was accomplished via the addition of 50% TCA at 1:1-1:3 volume–ratios, which resulted in a [TCA_final_] of 25.0, 33.3, and 37.5%, respectively. Immediately following blood collection, each rat was thoroughly perfused with 0.9% saline to rid the circulation of remaining dye, and the perfused brain and liver tissues were collected. For extraction, the brain and liver tissues were placed in 1:1–1:5 weight (mg):volume (µl) ratios of 50% TCA, and they were homogenized for 5 min (continuous beating) using a metal-bead homogenizer (BULLET BLENDER® BBX24). The TCA/extracts from the plasma, brain, and liver samples were centrifuged at 10,000 × *g* for 20 min to remove precipitates, tissue debris, and metal beads, and the supernatants were added to a 96-well plate (30 µl per well, each plate supplemented with 90 µl of 95% ethanol and thoroughly mixed by pipetting) for fluorescence spectroscopy (620 nm/680 nm).

### Animal care

The procedures that involved experimental animals were conducted in accordance with the Institutional Guidelines of China Medical University for the Care and Use of Experimental Animals (IGCMU-CUEA) and were approved by the Institutional Animal Care and Use Committee (IACUC) of China Medical University (Taichung, Taiwan) (Protocol No. 101-274-N). Male Sprague-Dawley rats (260–450 g) were used in this study. The rats had free access to rat chow and water *ad libitum* prior to use in the experiments.

### Data presentation and statistical analysis

The data are presented as the mean ± SEM. The extraction efficiencies by different volume-ratios of TCA were compared by 1-WAY or 2-WAY repeated measures ANOVA, in which the plasma samples and tissues from the same animals were matched. Each ANOVA test was followed by a Tukey's multiple comparisons test to determine the differences between specific groups.

## Author Contributions

H.L.W. and T.W.L. conceived the study and analyzed the data. H.L.W. performed the experiments. T.W.L. wrote the manuscript. Both authors reviewed and edited the manuscript.
